# Survey of liver pathologists to assess attitudes towards digital pathology and artificial intelligence

**DOI:** 10.1136/jcp-2022-208614

**Published:** 2023-01-04

**Authors:** Clare McGenity, Rebecca Randell, Christopher Bellamy, Alastair Burt, Alyn Cratchley, Robert Goldin, Stefan G Hubscher, Desley A H Neil, Alberto Quaglia, Dina Tiniakos, Judy Wyatt, Darren Treanor

**Affiliations:** 1 Pathology and Data Analytics, University of Leeds, Leeds, UK; 2 Department of Histopathology, Leeds Teaching Hospitals NHS Trust, Leeds, UK; 3 Faculty of Health Sciences, University of Bradford, Bradford, UK; 4 Wolfson Centre for Applied Health Research, Bradford, UK; 5 Division of Pathology, University of Edinburgh, Edinburgh, UK; 6 Translational and Clinical Research Institute, Newcastle University, Newcastle upon Tyne, UK; 7 Division of Digestive Diseases, Imperial College London, London, UK; 8 Institute of Immunology and Immunotherapy, University of Birmingham, Birmingham, UK; 9 Department of Cellular Pathology, Queen Elizabeth Hospital Birmingham, Birmingham, UK; 10 Department of Cellular Pathology, Royal Free Hospital, London, UK; 11 Translational and Clinical Research Institute, Newcastle University, Newcastle, UK; 12 Department of Pathology, National and Kapodistrian University of Athens, Athens, Greece; 13 Department of Clinical Pathology and Department of Clinical and Experimental Medicine, Linköping University, Linköping, Sweden; 14 Centre for Medical Image Science and Visualization (CMIV), Linköping University, Linköping, Sweden

**Keywords:** DIGITAL PATHOLOGY, LIVER DISEASE, HISTOPATHOLOGY, Image Processing, Computer-Assisted, COMPUTER SYSTEMS

## Abstract

**Aims:**

A survey of members of the UK Liver Pathology Group (UKLPG) was conducted, comprising consultant histopathologists from across the UK who report liver specimens and participate in the UK National Liver Pathology External Quality Assurance scheme. The aim of this study was to understand attitudes and priorities of liver pathologists towards digital pathology and artificial intelligence (AI).

**Methods:**

The survey was distributed to all full consultant members of the UKLPG via email. This comprised 50 questions, with 48 multiple choice questions and 2 free-text questions at the end, covering a range of topics and concepts pertaining to the use of digital pathology and AI in liver disease.

**Results:**

Forty-two consultant histopathologists completed the survey, representing 36% of fully registered members of the UKLPG (42/116). Questions examining digital pathology showed respondents agreed with the utility of digital pathology for primary diagnosis 83% (34/41), second opinions 90% (37/41), research 85% (35/41) and training and education 95% (39/41). Fatty liver diseases were an area of demand for AI tools with 80% in agreement (33/41), followed by neoplastic liver diseases with 59% in agreement (24/41). Participants were concerned about AI development without pathologist involvement 73% (30/41), however, 63% (26/41) disagreed when asked whether AI would replace pathologists.

**Conclusions:**

This study outlines current interest, priorities for research and concerns around digital pathology and AI for liver pathologists. The majority of UK liver pathologists are in favour of the application of digital pathology and AI in clinical practice, research and education.

**WHAT IS ALREADY KNOWN ON THIS TOPIC**

- Interest in the uses and potential of digital pathology and artificial intelligence (AI) has grown in recent years. These technologies will influence the diagnosis and management of liver diseases.

**WHAT THIS STUDY ADDS**

- This is the first study to examine the views of UK liver pathologists around digital pathology and AI and to highlight research priorities within liver pathology.

**HOW THIS STUDY MIGHT AFFECT RESEARCH, PRACTICE OR POLICY**

- The authors anticipate that this will influence the direction of research for digital pathology and AI applied to liver disease, by highlighting the demand and concerns from the UK liver pathology community.

## Introduction

Liver diseases are a major cause of global morbidity and mortality. Approximately two million deaths worldwide and over 150 000 across Europe are due to liver disease each year.^1 2^ Preventable deaths due to liver conditions amounted to 26 265 in England alone from 2015 to 2017.^3^ In liver disease, the histopathologist’s tissue assessment frequently plays an important role in determining diagnosis, prognosis and response to treatment for many conditions, especially where there is uncertainty in the patient’s clinical history and presentation.^4 5^ However, overall demand for pathology services is rising, the content of pathology reports is increasingly complex and histopathology laboratories face ongoing workforce challenges.^6 7^

Digital pathology is the digitisation of glass slides to make high-resolution images available for viewing on a computer screen.^8^ This is achieved through whole slide imaging, involving the use of scanners to capture an entire glass slide.^8^ Work is currently ongoing to deploy digital pathology to hospitals across the UK.^9^ Image analysis techniques ranging from conventional computerised morphology tools through to artificial intelligence (AI) have been used in pathology research for some years, although recent AI developments in deep learning have inspired researchers further.^10 11^ The increasing availability of digital pathology is bringing efforts to develop and clinically deploy AI tools closer to reality. In the face of the workforce and workflow challenges in pathology laboratories, the application of digital pathology and AI as tools to assist the histopathologist in reporting of liver specimens has generated substantial enthusiasm.^12–17^ However, understanding and targeting areas where most benefit can be achieved is important in addressing clinical demands and avoiding research waste.

The UK Liver Pathology Group (UKLPG) is a professional association that arose from the collaboration of several groups with liver pathology interests.^18^ They held their first meeting as the UKLPG in 2016 and its membership comprises consultant histopathologists (full members) from across the UK who report liver specimens in their clinical practice.^18^ The UKLPG is also responsible for running the UK liver pathology External Quality Assurance (EQA) scheme, and therefore, encompasses the vast majority of histopathologists who report liver cases from across the country. An academic subgroup of the UKLPG membership formed in 2020 to collaborate and focus on the development of digital pathology and AI for use in liver disease. Acknowledging the growing interest in this area, the group aimed to understand current attitudes and research priorities of UKLPG members for digital pathology and AI, and to report this to the wider research community.

## Methods

### Study design

A cross-sectional survey was developed by three members of the UKLPG (CM, JW and DTr) from February 2021 to April 2021. Broader consultation across a series of meetings with the digital pathology and AI academic subgroup of the UKLPG involved exploring experiences and opinions of group members, considering publications in this area and identifying areas of clinical utility for these technologies to inform the survey content. Further discussions with a qualitative researcher (RR) took place in helping to inform the design and content of the survey. The rationale for canvassing opinion from the UK liver pathology community and the full survey were reviewed and approved by the UKLPG committee prior to distribution. The authors are not aware of any studies attempting to gather opinion from the UK liver pathology community on the subject of digital pathology and AI prior to this exercise. The survey comprised a total of 50 questions, divided into 11 sections of multiple choice questions (MCQs) and 2 free text questions at the end to allow open responses. For MCQs addressing topics on digital pathology and AI, the answers were selected from a five point grading system from ‘strongly disagree’ to ‘strongly agree’ and there was an additional sixth option to state ‘don’t know’ if needed. The survey aimed to assess attitudes to digital pathology and AI for use in liver disease, respectively. The full text of the questionnaire is in online supplemental document 1.

10.1136/jcp-2022-208614.supp1 Supplementary data

### Survey distribution

The survey was circulated to all full members of the UKLPG via email (CM and JW) with a link to the SurveyMonkey online tool (www.surveymonkey.com) and included accompanying guidance for completion. It was sent to members in a routine mailing list email and was not mandatory to complete. Responses were collected from April 2021 to May 2021, and reminder emails and a deadline extension were given to encourage participation (CM and JW).

### Data analysis

Analyses for each question were performed using Microsoft Excel software (CM). Given the range of knowledge levels across the community, with some members having no experience and others with extensive experience of these technologies, none of the questions were mandatory. Some questions allowed more than one response to gather a wider reflection of participant experience. Therefore, the summary data are given on a per question basis. Despite this, responses ranged from totals of 40–42 responses per question from the total of 42 participants, as only a maximum of two respondents skipped any individual question.

### UKLPG membership involvement

Members of the UKLPG were involved throughout the conception of the work, study design, survey approval and distribution, analysis and interpretation of the data. Results of the survey were presented at the UKLPG annual meeting in December 2021, providing opportunity for further discussion, involvement and questions from the entire membership.

## Results

### Survey participants

A total of 42 of 116 consultant members of the UKLPG completed individual responses to the email survey, representing 36% of the full membership. Respondents were from a mixture of healthcare settings including transplant centres 26% (11/42), tertiary non-transplant centres 48% (20/42) and secondary care / district general hospitals 26% (11/42). Table 1 demonstrates participant characteristics by cases reported. The participant reporting rates of liver specimens varied from <20 per year (5%) to >500 per year (12%). All pathologists at transplant centres declared reporting at least two hundred liver cases per year, those at tertiary non-transplant centres reported between 20 and 500 cases and pathologists in secondary care/ district general hospitals (DGHs) reported between 1 and 500 cases. Response rates for all possible MCQ responses are shown by number of cases reported per year. Completion rate was 99% or more for all those who report 20 or more liver cases per year.

**Table 1:** Survey participant characteristics by case reporting numbers

No of cases reported per year	<20	20–49	50–199	200–500	>500
Total participant responses n (%)	2 (5)	10 (24)	15 (36)	10 (24)	5 (12)
Transplant centre n (%)	0 (0)	0 (0)	0 (0)	6 (14)	5 (12)
Tertiary non-transplant centre n (%)	0 (0)	4 (10)	13 (31)	3 (7)	0 (0)
Secondary care/District General Hospital (DGH) n (%)	2 (5)	6 (14)	2 (5)	1 (2)	0 (0)
Total response rate across all MCQs (%)	62	100	100	100	99

MCQs, multiple choice questions.

### Prior experience of digital pathology and AI

**Figure 1:**
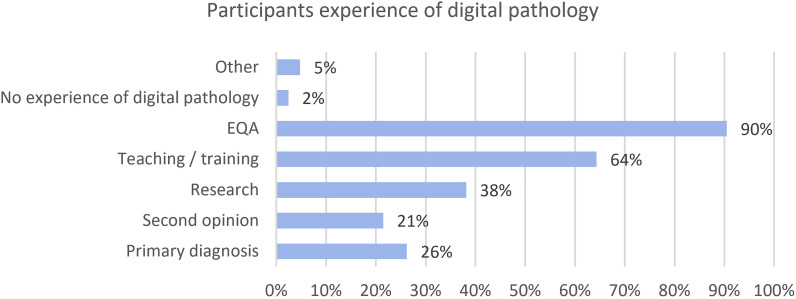
Responses for participants experiences of digital pathology for a range of purposes. EQA, external quality assurance.

Respondents were asked about their experience to date of using of digital pathology and AI. This included use in primary diagnosis, second opinion, research, teaching and EQA. Respondents could select multiple options and results are shown in figure 1. Ninety per cent (38/42) of participants had used digital pathology for EQA, followed by 64% (27/42) for teaching and training. Use for primary diagnosis and second opinion was reported in 26% (11/42) and 21% (9/42) of cases, respectively, and only one participant (2%, 1/42) had no experience of using digital pathology. Other examples in the comments included acquiring a scanner to trial the technology to assess where it may be useful. Of those reporting 200 or more liver cases per year, all had prior experience of digital pathology.

**Figure 2:**
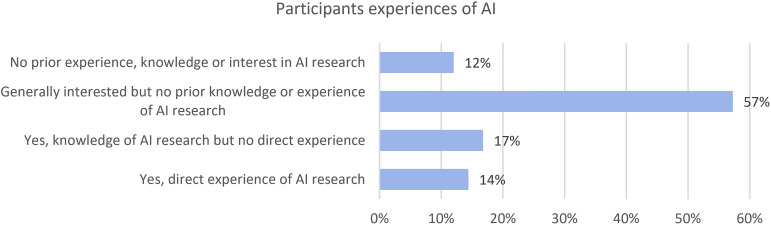
Responses for participants descriptions of their AI experience, knowledge and interest. AI, artificial intelligence.

As AI is not yet available for routine clinical use by UK histopathologists (apart from a few exceptions), questions on experience of AI focused on a research setting. Figure 2 outlines participants descriptions of their levels of experience with AI research. The majority of participants (57%) had no prior experience but had a general interest in AI development for histopathology. Twelve per cent of respondents had no prior experience, knowledge or interest in AI research.

### Digital pathology

**Figure 3:**
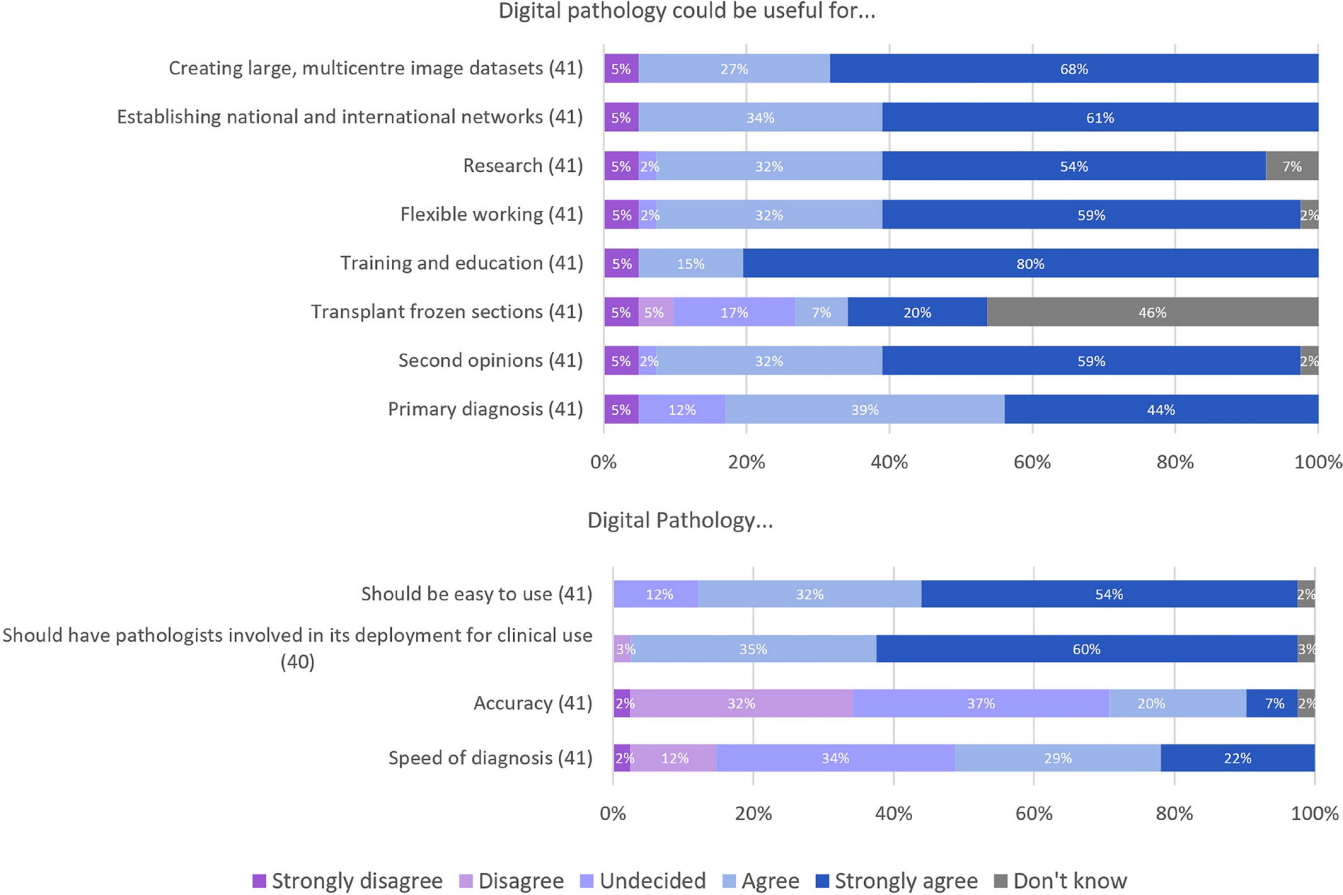
Responses for concepts around digital pathology.

Twelve uses and considerations for digital pathology were explored in this section of the survey, with responses shown in figure 3. For primary diagnosis of liver specimens, 83% (34/41) of respondents either agreed or strongly agreed that digital pathology could be useful. Ninety per cent (37/41) of respondents either agreed or strongly agreed that digital pathology could be useful for obtaining second opinions. Digital pathology was also identified as useful in both research and training/education, where 85% (35/41) and 95% (39/41) of respondents, respectively, either agreed or strongly agreed with its utility in these areas. There was less certainty when asked if digital pathology would improve accuracy of diagnosis, with the largest group of respondents at 37% (15/41) reporting to be undecided. However, flexible working was identified as a benefit of digital pathology with 90% of participants responding with ‘agree’ or ‘strongly agree’ when asked about this.

### Artificial intelligence

**Figure 4:**
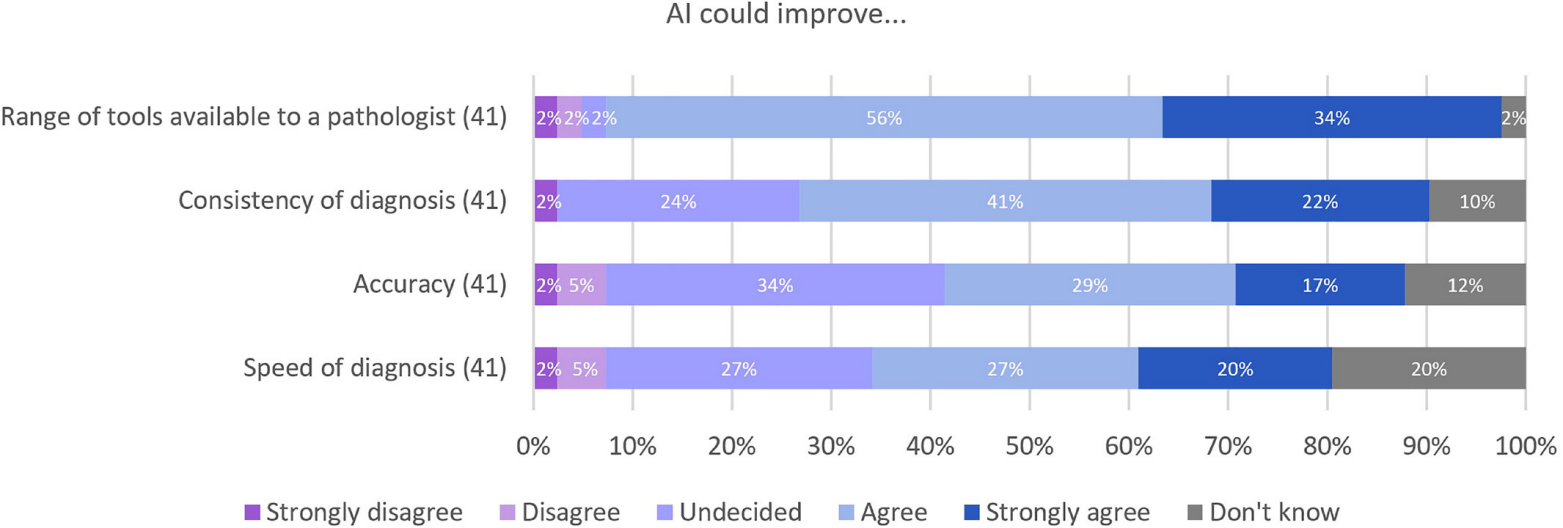
Responses for areas where AI may improve practice generally. AI, artificial intelligence.

**Figure 5:**
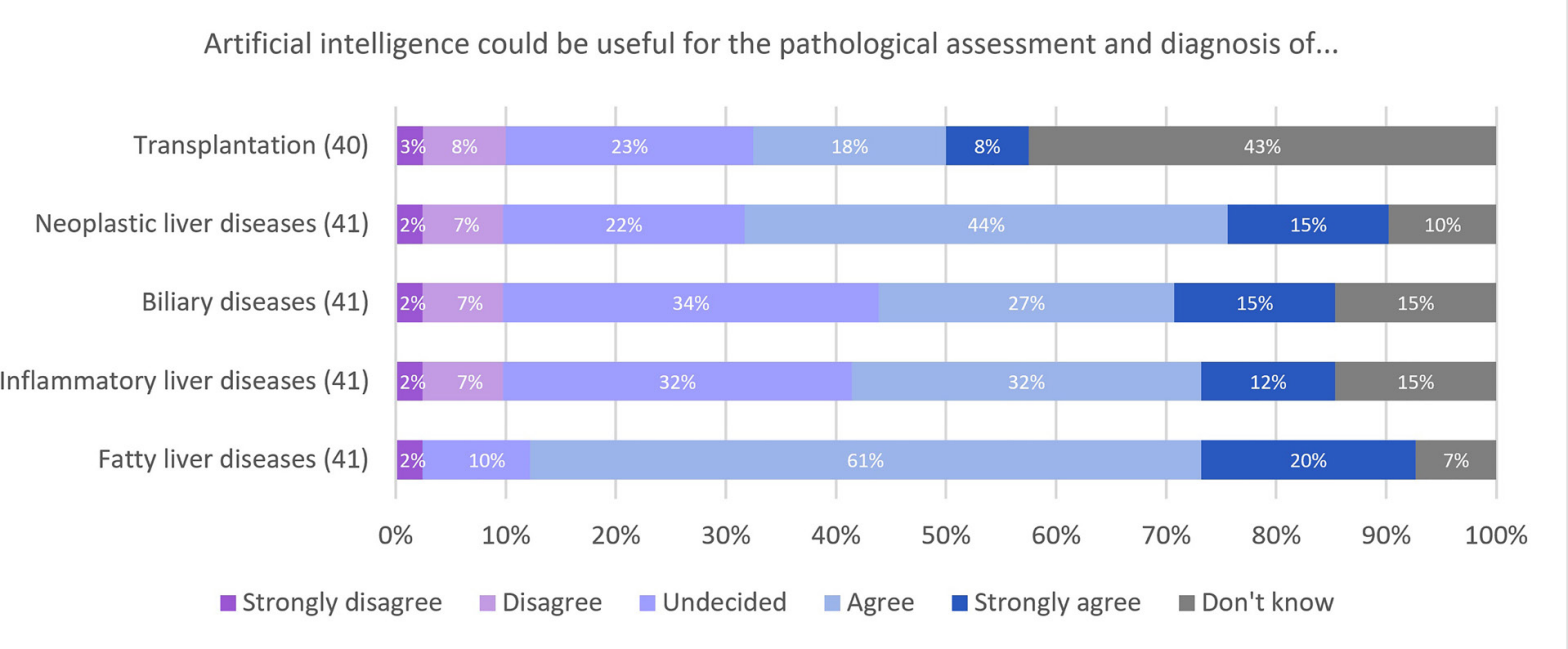
Responses for AI utility by disease group. AI, artificial intelligence.

**Figure 6:**
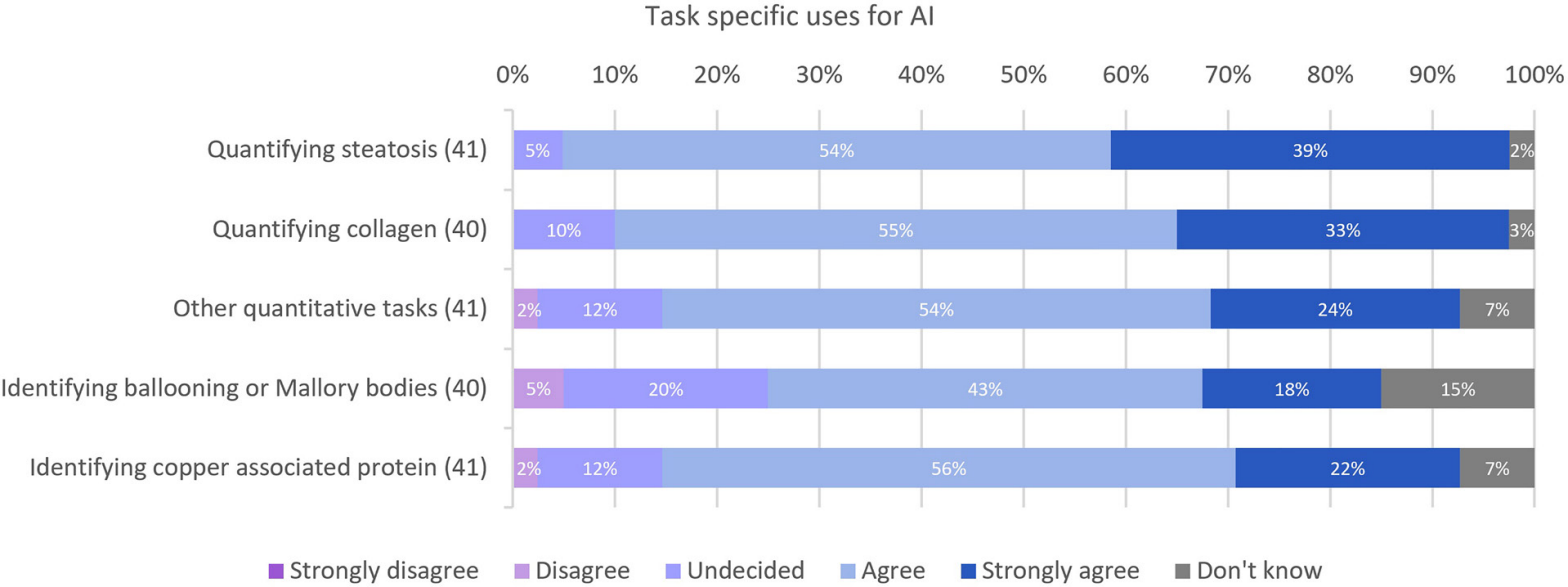
Responses for task specific uses for AI. AI, artificial intelligence.

Twenty-four topics were examined across four MCQs, exploring uses and potential of AI in pathology. Responses to many of these questions are shown across figures 4–6. It is not possible to include all questions within figures in the main paper. Therefore, questions with most agreement in the responses given are highlighted with the full responses are included in online supplemental document 2. For general concepts where AI may improve practice (figure 4), 90% (37/41) of respondents agreed or strongly agreed that AI could improve the range of tools available to the histopathologist in their clinical practice. Improving the consistency of diagnosis and the potential for understanding tissue features not currently recognised by pathologists, 63% (26/41) and 65% (26/40) of participants either agreed or strongly agreed with these concepts. Again, there was less certainty around the ability of AI to improve the speed or accuracy of diagnosis with 27% (11/41) and 34% (14/41) undecided.

10.1136/jcp-2022-208614.supp2 Supplementary data

When exploring the utility of AI by liver disease groups (figure 5), 80% (33/41) of participants identified fatty liver diseases as an area where AI could be useful for their clinical practice. Fifty-nine per cent (24/41) of participants either agreed or strongly agreed that AI would be useful for diagnosis and assessment of neoplastic liver diseases. There was less certainty for inflammatory liver diseases and biliary diseases, but still 44% (18/41) and 41% (17/41), respectively, of participants agreed or strongly agreed that AI would be useful in these contexts. Forty-three per cent (17/40) of participants responded ‘don’t know’ when asked about utility in transplantation.

Respondents were asked about 14 specific tasks where AI could potentially be applied in clinical practice. The list of tasks was derived from reviewing the literature and from the consultations prior to the design of the survey. The tasks where there was most recognised utility are shown in figure 6. Ninety-three per cent (38/41) and 88% (35/40) of participants agreed or strongly agreed that quantifying steatosis and quantifying collagen would be useful in their clinical practice, respectively. Other quantitative tasks (eg, counting bile duct or portal tract numbers), identifying copper associated protein and identifying ballooning or Mallory bodies were also of interest to the liver pathologists with 78% (32/41), 78% (32/41) and 60% (24/40), respectively, either agreeing or strongly agreeing that AI for these tasks would be useful. There was less certainty with tasks such as predicting patient outcomes in medical liver diseases and neoplastic diseases with responses in both cases at 41% (29/41) indicating that they were undecided. Although, identifying lymphovascular invasion was highlighted as another potentially useful task with 59% (24/41) of participants agreeing or strongly agreeing with this concept.

### Concerns

Eight areas of potential concern around the impact of AI on liver pathologists were explored. In this question, participants were most concerned about AI being developed for pathology laboratories without the involvement of a histopathologist, with 73% (30/41) responding ‘agree’ or ‘strongly agree’ when asked about this. This was followed by 63% (26/41) of respondents agreeing or strongly agreeing that AI may struggle with existing digital systems. However, when asked whether AI is likely to replace pathologists in the future, the majority were not concerned about this with 63% (26/41) responding ‘disagree’ or ‘strongly disagree’.

### Comments

At the end of the survey, two free-text questions asked participants to first highlight priority areas of digital pathology or AI research and second to make any other comments. Common themes in research priorities included quantitative tasks, fatty liver diseases, neoplastic liver diseases, improving variation between pathologists and practical benefits of digital pathology (eg, accessing archive cases, sharing images at meetings, collaboration and seeking second opinions). Comments were in keeping with the findings of the quantitative questions. Example quotes from the first free text question included: ‘help with grading of assessment’, ‘estimation of the amount of fatty change, fibrosis and inflammation’, ‘ALD/NAFLD (alcohol-related liver disease and non-alcoholic liver disease) when biopsies done’, ‘grading and typing of HCC’ (hepatocellular carcinoma), ‘reduction of inter/intraobserver variation’, ‘marking, measuring, photographing and use for teaching would all be improved’, ‘cross site collaboration’ and ‘national network so that digital slides can be viewed from anywhere’.

A range of points were raised in the final open question, with many expanding comments on the earlier questions and discussing benefits and concerns around the technologies. Examples of comments from this question were: ‘This is not only coming but has arrived. Pathologists have largely been resistant, indifferent or ignorant. We need to get ahead of the curve.’, ‘Should be done on a global basis and not piecemeal roll out’, ‘Requires pathologist input for development’, ‘The hype that advocates perpetuate in AI does everyone a disservice.’, ‘Digital pathology may not fulfil its potential for substantive pathologists, training and improving patient care’ and ‘If AI is widely adopted and proves to be useful, we need to rethink the specialty, training etc and be realistic about future’.

## Discussion

### Key findings

Recent developments in digital pathology and AI have impacted many areas of pathology, including liver disease.^10 19^ A survey published in 2018 examined the access and usage of digital pathology across UK pathology institutions, and this showed that there was great interest in digital pathology at that time.^20^ This survey also demonstrated that 41% of institutions were already using image analysis on digital slides in some capacity and there have been many developments in this field since the study was performed. While the 2018 survey was conducted at a more general and departmental level, and so direct comparison is difficult, it is notable that 60% of departments at this time had access to a digital pathology scanner but 58.8% of these reported that they did not produce any digital slides. Those who reported producing digital slides varied from 50 to 300 000 slides produced per year. This study of liver pathologists showed that only 2% (1/42) of participants had no experience of digital pathology and perhaps reflects wider general accessibility of digital slides compared with this earlier study. As well as uses of digital pathology for tasks such as primary diagnosis, second opinions and education; the availability of this technology facilitates the application of image analysis and AI.^10 21 22^ Image analysis techniques have been established in liver pathology research over several decades^23–26^; however, more recent AI techniques such as deep learning have generated high levels of interest and hold promise for future clinical applications.^13 19 27^

This study demonstrates the appeal and potential of digital pathology and AI to the clinical practice of liver pathologists. The majority of respondents had experience of using digital pathology and most were interested in the potential applications of AI. The usefulness of digital pathology for primary diagnosis, second opinions, research and teaching and training were all highlighted. As deployment of digital pathology is currently ongoing across the National Health Service (NHS), these findings emphasise current demand and recognition of utility within liver disease. Liver pathologists saw promise in AI expanding the range of diagnostic tools available to them and improving the consistency of diagnosis. However, there was uncertainty about its potential to improve the speed and accuracy of diagnosis. Although, it must be acknowledged that AI is not currently in use in the vast majority of laboratories, and so uncertainty around how it will function is not surprising. This was a brief snapshot survey of opinions around digital pathology and AI, intended to maximise participation from UK liver pathologists. It was therefore not possible to explore more extensive details such as precise numbers or proportions of digital cases reported, the digital pathology platforms used, the data storage solutions in place and the areas of AI research that some participants were working in, but these areas could be examined in future work. The comments illustrated some additional information about these topics, with one participant reporting that digital pathology was being ‘…implemented for primary rapid/urgent diagnosis and for second opinion’. Another reported trialling the use of digital pathology in principle. Several participants indicated issues with existing NHS information technology (IT) infrastructure as being potential barriers to using digital pathology for more cases. However, there were requests for digital pathology to be made available for use in a range of contexts included within the comments. There was also awareness of a variety of potential clinical applications of AI from many participants.

Fatty liver diseases and neoplastic liver diseases were determined as areas of demand for the application of AI, and many comments from participants discussed specific applications of AI for both of these disease groups. Transplantation was an area of uncertainty in terms of AI utility, however, this likely reflects that the majority of respondents (74%) do not practice routinely at transplant centres.

The task-specific questions and participant comments showed a focus on quantitative tools with the aim of removing some of these time consuming tasks from their practice. Identifying objects that may be difficult or laborious for a liver pathologist to find such as ballooning, Mallory bodies, copper associated protein and vascular invasion were highlighted as well. Participants were undecided regarding the potential of AI to predict patient outcomes. This may reflect the increased complexity of this task when compared with simple quantitation or the existence of alternative tools in place to do this.

Key concerns to acknowledge were the potential lack of involvement of pathologists in AI development and the implementation of AI tools within existing digital systems and infrastructure within laboratories. These are concerns that will need to be addressed to achieve future successful implementation of AI products. Finally, it is of note that the majority of liver pathologists are not concerned (63%) that AI could replace them in the future.

### Study limitations

While the response rate (36%) was good for an unsolicited email survey, there is a possible non-response bias when accounting for members who did not participate. To reflect the varying knowledge levels of the participants, not all questions were mandatory and so responses ranged from 40 to 42 responses per question from the 42 participants, and a non-response bias is possible here. It should be acknowledged that current variation in levels of experience and exposure to digital pathology and AI may have influenced responses both positively or negatively. This survey provides a useful snapshot of opinion at one point in time, and this may change with rapid advancement of these technologies. A formal Delphi exercise could be explored in the future to follow this initial piece of work and to expand on the themes identified here.

## Conclusion

Given the current pace of change on a national scale in the UK, with increasing availability of digital pathology solutions and a growing body of AI research, this survey aimed to gather current attitudes and priorities around both of these technologies. Demand for digital pathology access and numerous applications of AI for tasks to aid the liver pathologist in clinical practice were identified. Findings highlighted within this study could be used to help inform the direction of future research within this field, to outline current areas of need and also potential concerns when implementing these tools.

## Data Availability

All data relevant to the study are included in the article or uploaded as online supplemental information.
