# Supplementary material for: Survey of liver pathologists to assess attitudes towards digital pathology and artificial intelligence
Source: J Clin Pathol. 2023 Jan 4;77(1):27–33. doi: 10.1136/jcp-2022-208614 (PMC10804041; doi:10.1136/jcp-2022-208614)
Supplement: Supplementary data [file jcp-2022-208614supp001.pdf]

United Kingdom Liver Pathology Group

Survey for members of UKLPG: Digital Pathology and AI in liver disease

Introduction

Thank you for participating in this survey. Your responses will build a picture of the UK liver pathology community's opinions on digital pathology and artificial intelligence (AI) and we anticipate the results will be published as a report. This report could influence future decisions about research priorities in this area. This survey aims to build on discussions from a focus group of interested pathologists working in liver research. Your individual answers will remain confidential throughout this process and will not be used for purposes other than those outlined above.

This first page contains some general introductory questions that we would like all participants to complete. The following pages are optional but we would be grateful for your insight. If you do not feel able to answer certain questions, please select "don't know" or you can skip to the next question.

* 1. Describe the hepatology services in your current place of work

- ◯ Tranplant centre
- ◯ Tertiary non-transplant centre
- ◯ Secondary care / DGH
- ◯ Other (please specify):

McGenity C, *et al. J Clin Pathol* 2023; 77:27–33. doi: 10.1136/jcp-2022-208614

* 2. Approximately how many liver specimens would you typically report per year

- <20
- 20-49
- 50-199
- 200-500
- >500

* 3. What is your experience of using digital pathology? (Please select all those that apply)

- Primary diagnosis
- Second opinion
- Researc h
- Teaching / training
- EQA
- No experience of digital pathology
- Other (please specify)

* 4. Do you have any experience, knowledge or interest in AI development? (Please select the best description)

- Yes, direct experience of AI research
- Yes, knowledge of AI research but no direct experience
- Generally interested but no prior knowledge or experience of AI research
- No prior experience, knowledge or interest in AI research

United Kingdom Liver Pathology Group

Survey for members of UKLPG: Digital Pathology and AI in liver disease

Digital Pathology

Digital pathology usually refers to images of histology generated by whole slide imaging and available to review by a pathologist on a computer.

Access and experience may vary across the group, but we would like to understand what considerations are important to liver pathologists in this area. With your current knowledge and future expectations, please rate how the questions relate to you.

Questions are rated from strongly disagree to strongly agree, with the option to say "don't know" or you may skip the question. Comments and suggestions may be added at the bottom of each question.

McGenity C, *et al. J Clin Pathol* 2023; 77:27–33. doi: 10.1136/jcp-2022-208614

5. **Digital pathology** could be useful for:

	Strongly disagree	Disagree	Undecided	Agree	Strongly agree	Don't know
Primary diagnosis	◯	◯	◯	◯	◯	◯
Second opinions	◯	◯	◯	◯	◯	◯
Transplant frozen sections	◯	◯	◯	◯	◯	◯
Training and education	◯	◯	◯	◯	◯	◯
Flexible working	◯	◯	◯	◯	◯	◯
Research	◯	◯	◯	◯	◯	◯
Establishing national and international networks	◯	◯	◯	◯	◯	◯
Creating large, multicentre image datasets	◯	◯	◯	◯	◯	◯

Additional comments / suggestions

6. **Digital pathology:**

	Strongly disagree	Disagree	Undecided	Agree	Strongly agree	Don't know
Could improve speed of diagnosis						
Could improve accuracy of diagnosis						
Should have pathologists involved in its deployment for clinical use						
Should be easy to use						

Additional comments / suggestions

United Kingdom Liver Pathology Group

Survey for members of UKLPG: Digital Pathology and AI in liver disease

Artificial intelligence (general concepts)

Artificial intelligence usually refers to machines demonstrating intelligent behaviour by evaluating their environment and taking resulting actions. In the context of histopathology, this is often referring to machines extracting information from an image.

Access and experience may vary across the group but we would like to understand what considerations in general are important to liver pathologists in this area. With your current knowledge and future expectations, please rate how the questions relate to you.

7. **Artificial intelligence could improve:**

	Strongly disagree	Disagree	Undecided	Agree	Strongly agree	Don't know
Speed of diagnosis	◯	◯	◯	◯	◯	◯
Accuracy of diagnosis	◯	◯	◯	◯	◯	◯
Consistency of diagnosis	◯	◯	◯	◯	◯	◯
The range of tools available to a pathologist	◯	◯	◯	◯	◯	◯
Understanding of tissue features not currently recognised by pathologists	◯	◯	◯	◯	◯	◯

Additional comments / suggestions

United Kingdom Liver Pathology Group

Survey for members of UKLPG: Digital Pathology and AI in liver disease

Artificial intelligence and liver diseases

We would like to establish where AI might be useful to assist with diagnostic work. Please rate how useful you think AI tools would be in assessment and diagnosis these diseases.

McGenity C, *et al. J Clin Pathol* 2023; 77:27–33. doi: 10.1136/jcp-2022-208614

8. **Artificial intelligence** could be useful for the pathological assessment and diagnosis of

	Strongly disagree	Disagree	Undecided	Agree	Strongly agree	Don't know
Fatty liver diseases	◯	◯	◯	◯	◯	◯
Inflammatory liver diseases	◯	◯	◯	◯	◯	◯
Biliary diseases	◯	◯	◯	◯	◯	◯
Neoplastic liver diseases	◯	◯	◯	◯	◯	◯
Transplantation	◯	◯	◯	◯	◯	◯

Additional comments / suggestions

United Kingdom Liver Pathology Group

Survey for members of UKLPG: Digital Pathology and AI in liver disease

Artificial intelligence tools

We have suggested some potential tools that might be performed by a computer with an AI algorithm to assist with your diagnostic work. Please rate whether these would be useful.

Possible concerns are included at the bottom, please rate whether or not these are of concern to you.

9. Artificial intelligence tools performing the following for diagnostic work would be useful in **medical liver diseases**

	Strongly disagree	Disagree	Undecided	Agree	Strongly agree	Don't know
Quantifying steatosis	◯	◯	◯	◯	◯	◯
Quantifying collagen	◯	◯	◯	◯	◯	◯
Other quantitative tasks e.g. bile duct or portal tract numbers	◯	◯	◯	◯	◯	◯
Identifying inflammation	◯	◯	◯	◯	◯	◯
Identifying ballooning or Mallory bodies	◯	◯	◯	◯	◯	◯
Identifying copper associated protein	◯	◯	◯	◯	◯	◯
Predicting patient outcomes	◯	◯	◯	◯	◯	◯

Additional comments / suggestions

McGenity C, *et al. J Clin Pathol* 2023; 77:27–33. doi: 10.1136/jcp-2022-208614

10. Artificial intelligence tools performing the following for diagnostic work would be useful in **neoplastic liver disease**

	Strongly disagree	Disagree	Undecided	Agree	Strongly agree	Don't know
Quantify tumour grade	◯	◯	◯	◯	◯	◯
Identifying lymphovascular invasion	◯	◯	◯	◯	◯	◯
Identifying perineural invasion	◯	◯	◯	◯	◯	◯
Classifying nodule type e.g. regenerative, dysplastic, HCC	◯	◯	◯	◯	◯	◯
Providing morphomolecular classification	◯	◯	◯	◯	◯	◯
Predicting patient outcomes	◯	◯	◯	◯	◯	◯
Predicting genetics	◯	◯	◯	◯	◯	◯

Additional comments / suggestions

11. I am concerned that **artificial Intelligence** may:

	Strongly disagree	Disagree	Undecided	Agree	Strongly agree	Don't know
Be developed without pathologist involvement	○	○	○	○	○	○
Replace pathologists	○	○	○	○	○	○
Make decisions that we cannot understand	○	○	○	○	○	○
Create additional work for the pathologist	○	○	○	○	○	○
Impair training	○	○	○	○	○	○
De-skill pathologists	○	○	○	○	○	○
Be unsafe for patients	○	○	○	○	○	○
Struggle with existing digital systems	○	○	○	○	○	○

Additional comments / suggestions

United Kingdom Liver Pathology Group

Survey for members of UKLPG: Digital Pathology and AI in liver disease

Priorities and final comments

Please outline any concept(s) that you feel would be most important or useful to your practice in this area of research. Please feel free to add any final comments or suggestions.

10

Thank you for completing this survey.

12. If you were to highlight a priority / priorities for research and development in this area that would be most useful for your practice, what would you choose?

13. Please give any further comments or additional suggestions

McGenity C, *et al. J Clin Pathol* 2023; 77:27–33. doi: 10.1136/jcp-2022-208614
