# Supplementary material for: Survey of liver pathologists to assess attitudes towards digital pathology and artificial intelligence
Source: J Clin Pathol. 2023 Jan 4;77(1):27–33. doi: 10.1136/jcp-2022-208614 (PMC10804041; doi:10.1136/jcp-2022-208614)
Supplement: Supplementary data [file jcp-2022-208614supp002.pdf]

Supplementary document 2 – Summary of responses

Responses tables are given as number of responses and percentage of responses for each question.

Q1 Describe the hepatology services in your current place of work

Transplant centre	Tertiary non-transplant centre	Secondary care / DGH	Other	Total skipped
11	20	11	0	0

Transplant centre	Tertiary non-transplant centre	Secondary care / DGH	Other
26%	48%	26%	0%

Q2 Approximately how many liver specimens would you typically report per year

<20	20-49	50-199	200-500	>500	Total skipped
2	10	15	10	5	0

<20	20-49	50-199	200-500	>500
5%	24%	36%	24%	12%

Q3 What is your experience of using digital pathology

Primary diagnosis	Second opinion	Research	Teaching / training	EQA	No experience of digital pathology	Other	Total skipped
11	9	16	27	38	1	2	0

Primary diagnosis	Second opinion	Research	Teaching / training	EQA	No experience of digital pathology	Other
26%	21%	38%	64%	90%	2%	5%

Q4 Do you have any experience, knowledge or interest in AI development?

Yes, direct experience of AI research	Yes, knowledge of AI research but no direct experience	Generally interested but no prior knowledge or experience of AI research	No prior experience, knowledge or interest in AI research
6	7	24	5

Yes, direct experience of AI research	Yes, knowledge of AI research but no direct experience	Generally interested but no prior knowledge or experience of AI research	No prior experience, knowledge or interest in AI research
14%	17%	57%	12%

Q5 Digital pathology could be useful for

	Strongly disagree	Disagree	Undecided	Agree	Strongly agree	Don't know	Total skipped
Primary diagnosis	2	0	5	16	18	0	1
Second opinions	2	0	1	13	24	1	1
Transplant frozen sections	2	2	7	3	8	19	1
Training and education	2	0	0	6	33	0	1
Flexible working	2	0	1	13	24	1	1
Research	2	0	1	13	22	3	1
Establishing national and international networks	2	0	0	14	25	0	1
Creating large, multicentre image datasets	2	0	0	11	28	0	1

	Strongly disagree	Disagree	Undecided	Agree	Strongly agree	Don't know
Primary diagnosis	5%	0%	12%	39%	44%	0%
Second opinions	5%	0%	2%	32%	59%	2%
Transplant frozen sections	5%	5%	17%	7%	20%	46%
Training and education	5%	0%	0%	15%	80%	0%
Flexible working	5%	0%	2%	32%	59%	2%
Research	5%	0%	2%	32%	54%	7%
Establishing national and international networks	5%	0%	0%	34%	61%	0%
Creating large, multicentre image datasets	5%	0%	0%	27%	68%	0%

Q6 Digital pathology:

	Strongly disagree	Disagree	Undecided	Agree	Strongly agree	Don't know	Total skipped
Could improve speed of diagnosis	1	5	14	12	9	0	1
Could improve accuracy of diagnosis	1	13	15	8	3	1	1
Should have pathologists involved in its deployment for clinical use	0	1	0	14	24	1	2
Should be easy to use	0	0	5	13	22	1	1

	Strongly disagree	Disagree	Undecided	Agree	Strongly agree	Don't know
Could improve speed of diagnosis	2%	12%	34%	29%	22%	0%
Could improve accuracy of diagnosis	2%	32%	37%	20%	7%	2%
Should have pathologists involved in its deployment for clinical use	0%	3%	0%	35%	60%	3%
Should be easy to use	0%	0%	12%	32%	54%	2%

Q7 Artificial intelligence could improve:

	Strongly disagree	Disagree	Undecided	Agree	Strongly agree	Don't know	Total skipped
Speed of diagnosis	1	2	11	11	8	8	1
Accuracy	1	2	14	12	7	5	1
Consistency of diagnosis	1	0	10	17	9	4	1
The range of tools available to a pathologist	1	1	1	23	14	1	1
Understanding of tissue features not currently recognised by pathologists	1	2	8	15	11	3	2

	Strongly disagree	Disagree	Undecided	Agree	Strongly agree	Don't know
Speed of diagnosis	2%	5%	27%	27%	20%	20%
Accuracy	2%	5%	34%	29%	17%	12%
Consistency of diagnosis	2%	0%	24%	41%	22%	10%
Range of tools available to a pathologist	2%	2%	2%	56%	34%	2%
Understanding of tissue features not currently recognised by pathologists	3%	5%	20%	38%	28%	8%

Q8 Artificial intelligence could be useful for the pathological assessment and diagnosis of

	Strongly disagree	Disagree	Undecided	Agree	Strongly agree	Don't know	Total skipped
Fatty liver diseases	1	0	4	25	8	3	1
Inflammatory liver diseases	1	3	13	13	5	6	1
Biliary diseases	1	3	14	11	6	6	1
Neoplastic liver diseases	1	3	9	18	6	4	1
Transplantation	1	3	9	7	3	17	2

	Strongly disagree	Disagree	Undecided	Agree	Strongly agree	Don't know
Fatty liver diseases	2%	0%	10%	61%	20%	7%
Inflammatory liver diseases	2%	7%	32%	32%	12%	15%
Biliary diseases	2%	7%	34%	27%	15%	15%
Neoplastic liver diseases	2%	7%	22%	44%	15%	10%
Transplantation	3%	8%	23%	18%	8%	43%

Q9 Artificial intelligence tools performing the following for diagnostic work would be useful in medical liver diseases

	Strongly disagree	Disagree	Undecided	Agree	Strongly agree	Don't know	Total skipped
Quantifying steatosis	0	0	2	22	16	1	1
Quantifying collagen	0	0	4	22	13	1	2
Other quantitative tasks e.g. bile duct or portal tract numbers	0	1	5	22	10	3	1
Identifying inflammation	0	3	11	16	4	7	1
Identifying ballooning or Mallory bodies	0	2	8	17	7	6	2
Identifying copper associated protein	0	1	5	23	9	3	1
Predicting patient outcomes	0	0	17	12	2	10	1

	Strongly disagree	Disagree	Undecided	Agree	Strongly agree	Don't know
Quantifying steatosis	0%	0%	5%	54%	39%	2%
Quantifying collagen	0%	0%	10%	55%	33%	3%
Other quantitative tasks	0%	2%	12%	54%	24%	7%
Identifying inflammation	0%	7%	27%	39%	10%	17%
Identifying ballooning or Mallory bodies	0%	5%	20%	43%	18%	15%
Identifying copper associated protein	0%	2%	12%	56%	22%	7%
Predicting patient outcomes	0%	0%	41%	29%	5%	24%

Q10 Artificial intelligence tools performing the following for diagnostic work would be useful in neoplastic liver disease

	Strongly disagree	Disagree	Undecided	Agree	Strongly agree	Don't know	Total skipped
Quantifying tumour grade	0	3	10	16	5	7	1
Identifying lymphovascular invasion	0	2	8	18	6	7	1
Identifying perineural invasion	0	1	11	15	8	6	1
Classifying nodule type e.g. regenerative, dysplastic, HCC	0	3	15	10	4	8	2
Providing morphomolecular classification	0	2	12	17	5	5	1
Predicting patient outcomes	0	0	17	12	3	9	1
Predicting genetics	0	0	16	12	2	10	2

	Strongly disagree	Disagree	Undecided	Agree	Strongly agree	Don't know
Quantifying tumour grade	0%	7%	24%	39%	12%	17%
Identifying lymphovascular invasion	0%	5%	20%	44%	15%	17%
Identifying perineural invasion	0%	2%	27%	37%	20%	15%
Classifying nodule type e.g. regenerative, dysplastic, HCC	0%	8%	38%	25%	10%	20%
Providing morphomolecular classification	0%	5%	29%	41%	12%	12%
Predicting patient outcomes	0%	0%	41%	29%	7%	22%
Predicting genetics	0%	0%	40%	30%	5%	25%

Q11 I am concerned that artificial intelligence may:

	Strongly disagree	Disagree	Undecided	Agree	Strongly agree	Don't know	Total skipped
Be developed without pathologist involvement	0	9	1	20	10	1	1
Replace pathologists	8	18	5	6	2	2	1
Make decisions that we cannot understand	0	8	11	11	5	6	1
Create additional work for the pathologist	0	9	9	16	4	3	1
Impair training	2	12	11	9	4	3	1
De-skill pathologists	3	13	11	11	3	0	1
Be unsafe for patients	2	13	12	9	2	3	1
Struggle with existing digital systems	1	3	6	18	8	5	1

	Strongly disagree	Disagree	Undecided	Agree	Strongly agree	Don't know
Be developed without pathologist involvement	0%	22%	2%	49%	24%	2%
Replace pathologists	20%	44%	12%	15%	5%	5%
Make decisions that we cannot understand	0%	20%	27%	27%	12%	15%
Create additional work for the pathologist	0%	22%	22%	39%	10%	7%
Impair training	5%	29%	27%	22%	10%	7%
De-skill pathologists	7%	32%	27%	27%	7%	0%
Be unsafe for patients	5%	32%	29%	22%	5%	7%
Struggle with existing digital systems	2%	7%	15%	44%	20%	12%
